# Research on the application of refinement hierarchical population algorithm in MPPT of photovoltaic arrays

**DOI:** 10.1371/journal.pone.0342496

**Published:** 2026-02-18

**Authors:** Wei Liming, Kou Qi

**Affiliations:** School of Electrical and Computer Engineering, Jilin Jianzhu University, Changchun, Jilin, China; Aalto University, FINLAND

## Abstract

Considering the slow convergence speed and unstable transmission capacity of photovoltaic systems in maximum power point tracking (MPPT), this paper proposes a MPPT control strategy based on the fine-layered population algorithm. This strategy improves the search efficiency of the algorithm by fine-layered sequence reconstruction of the sparrow search algorithm (SSA) and particle swarm optimization algorithm (PSO) populations. Firstly, a PSO population pause mechanism is introduced to avoid the premature convergence problem of PSO. Secondly, a fine-layered sequence is used to generate a uniformly distributed initial population, and the cooperative optimization of the sparrow group and the particle swarm is designed to improve the compatibility of the algorithm. The two main populations are decomposed, the number of individuals in the population is optimized, and the individual optimal fitness value of the population is updated. At the same time, the population position is reorganized in each layer to enable each layer to optimize independently, making the entire population easier to collaborate, increasing population diversity, and thereby improving search efficiency. Finally, strategies such as filling functions and sign matrices are introduced into the speed update position of the improved hierarchical population algorithm (PSSSA) to reduce the adverse effects of power fluctuations caused by boundary effects, improve the convergence and speed of the algorithm, and ensure that the optimal solution is obtained after each update. To verify the effectiveness of the PSSSA algorithm, a simulation model under the weather conditions of a certain laboratory in Northeast China was constructed using Matlab. The simulation results show that when it is applied to the maximum power tracking problem, in the case of rapid changes in external environmental illumination, it can accurately track the maximum power point within 0.2 seconds. Its tracking stability is significantly better than the Particle Swarm Optimization (PSO) algorithm, the Seagull Search Algorithm (SSA), and the Perturbation Observation (P&O) algorithm. The size of the maximum power point is approximately 64% larger than that of the PSO algorithm. At the same time, it can also ensure the stability and rapidity of the tracking effect.

## Introduction

With the rapid depletion of traditional energy sources, the development and utilization of new energy sources have also received widespread attention. The traditional Maximum power point tracking methods such as CVT, INC and the Perturbation and Observation (P&O) method cannot solve this problem. With the development of AI and artificial intelligence technologies, technologies such as biological neural networks, artificial neural networks (ANNs), and Fuzzy Logic Control have permeated various fields of electrical engineering and have been applied to the MPPT control technology of photovoltaic power generation.

When the traditional maximum power point tracking (MPPT) strategies have limitations, in recent years, methods combining artificial intelligence have been proposed to enhance the maximum power point [1]. The main approaches include the combination method for wind power generation fields [2]. There are also strategies that integrate algorithms and consider multiple state parameters to improve MPPT [3,4]. The MSSSA algorithm demonstrates excellent optimization performance. This algorithm has also been successfully applied to two engineering optimization problems, proving the superiority of MSSSA in solving practical problems [5]. Among them, an adaptive mechanism is adopted to adjust weights and learning coefficients, achieving a balance between the tracking speed and accuracy of power output (P&O) [6]. The balance method is used to improve the level of photovoltaic systems, and the conversion efficiency of photovoltaic power generation has also been improved [7]. The most famous way of combining the particle swarm optimization algorithm with other algorithms has improved it, effectively reducing power loss caused by step size oscillation during the search process, thereby improving the conversion efficiency of photovoltaic power generation [8–10]. The Newton interpolation method is used to improve the search accuracy of the algorithm in the global optimum and increase the power generation efficiency [11]. The minimum mean square (LMS) controller using adaptive linear neurons (ADALINE) is used for reactive power compensation [12]. An improved TLIC method with delay support based on matching FRC peak and valley times [13]. The FPPT method is applied to make the maximum power respond in the grid [14]. The current prediction control method is applied within the traditional P&O method to achieve MPPT in three-phase power generation systems [15]. The regression algorithm is combined with the particle swarm optimization algorithm to improve MPPT [16]. The auxiliary frequency and adaptive frequency control methods are used to improve the maximum power point tracking technology [17,18]. Of course, the PSO, ABC, and grey wolf optimization algorithms for MPPT of photovoltaic arrays have been compared, and the tracking errors are all less than 0.5% [19]. The grid-connected photovoltaic inverter with island protection function and intelligent algorithms are used to improve the efficiency of MPPT in the grid [20,21]. At the same time, the convergence accuracy and optimization efficiency of the whale particle swarm fusion algorithm are improved, achieving the complementary advantages of the hybrid algorithm, and the convergence efficiency of the whale algorithm is also improved in the later stage [22]. In recent years, the integration of algorithms has become an important research direction in the MPPT field. Among them, the hybrid algorithm of particle swarm optimization (PSO) and grey wolf optimization (GWO) has stood out, demonstrating significant advantages in improving power convergence speed, tracking accuracy, and reducing the amplitude of power oscillations during the iterative process [23]. In response to the inherent deficiencies of intelligent swarm algorithms, such as the whale optimization algorithm (WOA), which, despite its strong global search capability and excellent performance in mechanical fault signal decomposition [24], has relatively weak local search ability; and the sparrow search algorithm (SSA), which, with its outstanding local search characteristics, plays a key role in short-term photovoltaic power generation prediction [25]. Research shows that combining the advantages of WOA and SSA to construct a hybrid optimization algorithm has led to significant improvements in the evaluation indicators of relevant models for wind power prediction [26]. These research results fully demonstrate the important value and application potential of hybrid algorithms in the MPPT field.

In conclusion, numerous studies have been applied in the literature. Overall, various characteristics have been investigated. The advantages and disadvantages of the MPPT method in the current literature are detailed in Table 1.

**Table 1:** Comparison of MPPT methods.

MPPT Algorithm	Merits	Demerits
MSSSA control MPPT [5]	Applied to two engineering optimization problems	Large number of steps involved, special parameter selection is necessary to handle the processing load.
RG-PSO [16]	The local search ability is relatively weak.	The processing load generated by the need to continuously calculate the number of steps.
WOA-PSO [22]	Improve convergence accuracy and optimize efficiency	Special equipment may be required to accommodate the space conditions.
GRO-SSA-LSTM [25]	The calculation time is short, the computational complexity is low, and the performance is stable.	The computational complexity is high and requires specific parameter selection.
WOA-VMD-SSA-LSTM [26]	MPP features low oscillation, low overshoot and high performance.	The processing load resulting from the continuous calculation of steps.

Realizing rapid and accurate maximum power point tracking under local shading conditions remains a significant challenge for photovoltaic systems. The existing algorithms still have room for improvement in terms of convergence speed and dynamic stability. Therefore, this study proposes an innovative PSSSA fusion algorithm aimed at significantly enhancing the MPPT performance of photovoltaic systems in complex local shading environments. This algorithm initializes the population through a refined hierarchical sequence strategy, generating a uniformly distributed initial population for PSO and SSA, and introduces a dynamic nonlinear fusion mechanism based on continuous diagnosis of the search state. This mechanism can adaptively adjust the proportion of PSO and SSA strategies according to the real-time search state, achieving faster convergence among multiple power peaks and effectively suppressing steady-state oscillations. Additionally, this study constructs an expandable meta-heuristic framework that integrates strategies such as symbolic matrices, forming a comprehensive control method that balances tracking accuracy, convergence speed, and operational stability. This provides a systematic solution to address the adaptability of population algorithms in multi-peak MPPT tasks. The structure of the remaining parts of this paper is as follows. The first part introduces the system framework and the modeling of MPPT working strategies. The second part elaborates on the basic control strategies of the proposed algorithm. Moreover, the third part will present the simulation results supporting the proposed control strategies. Finally, the fourth part provides the conclusion.

## Photovoltaic system array model

**Fig 1:**
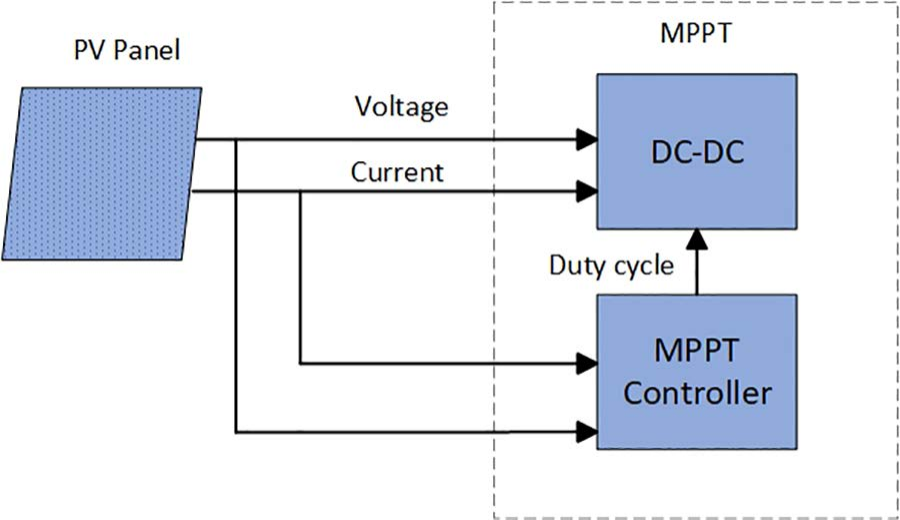
Photovoltaic array module.

A photovoltaic array is a semiconductor device that directly converts sunlight into electrical energy. It is the phenomenon where a PN junction generates an electric potential under illumination. Based on the analysis of sunlight in the Northeast region and combined with the basic situation of the laboratory, this paper selects a 3 × 1 photovoltaic array composed of three photovoltaic modules connected in series as an example for modeling under standard test conditions. The circuit layout of the Block Parameters PV photovoltaic panel is shown in Fig 1.

The volt-ampere characteristic curve of a photovoltaic cell is the superposition of the volt-ampere curve of a diode in the dark and the photocurrent, Assuming $R_{s}\ll R_{sh}$ then $R_{s}$=0。The equation of the volt-ampere curve is


$$I=I_{ph}-I_{\text{0}}\left[exp\left(\frac{qU}{nKT}\right)-\text{1}\right]$$ (1)

In the formula, n represents the ideal coefficient of the diode; k is the Boltzmann constant; T is the absolute temperature; q is the unit charge quantity, $I_{ph}$is the photocurrent, $I_{\text{0}}$ is the current flowing through the diode. When both the parallel resistance and the series resistance exist, the relationship between the current I and the voltage U of the photovoltaic cell is


$$I=I_{ph}-I_{\text{0}}\{exp\left[\frac{q\left(U+IR_{s}\right)}{nKT}\right]\;\;\;\;-\text{1}\}-\frac{U+IR_{s}}{R_{sh}}$$ (2)

**Fig 2:**
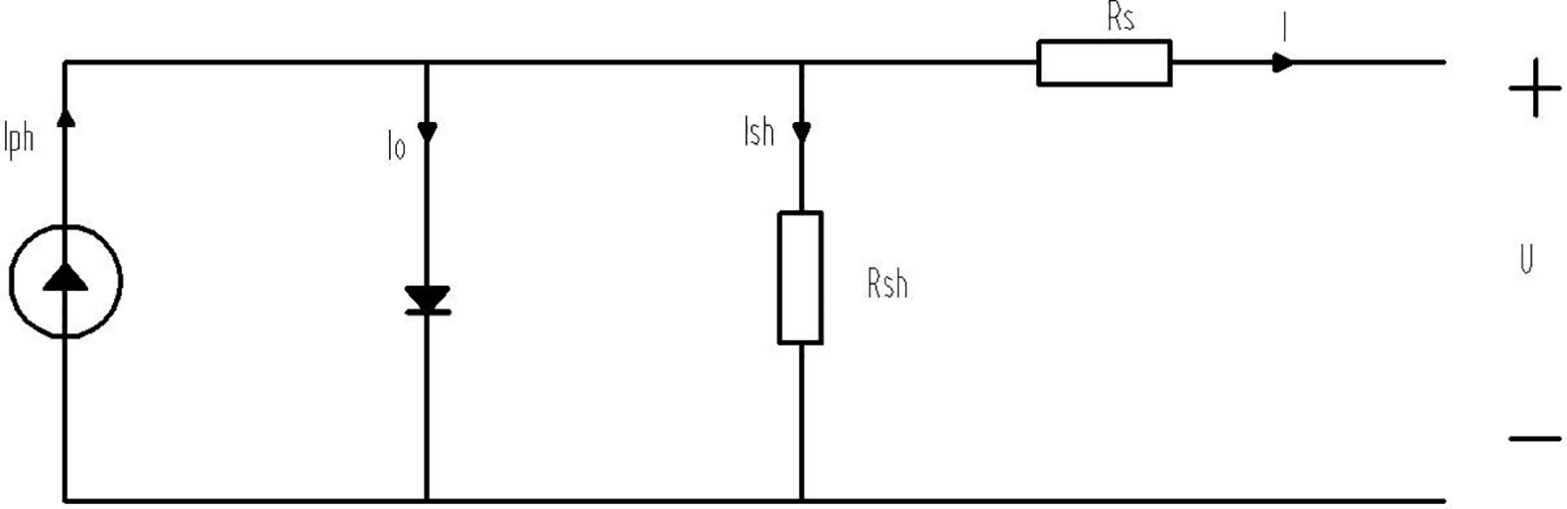
Equivalent Model of Photovoltaic Cell.

Fig 2 shows a simple model of a photovoltaic cell. In the figure, $I_{ph}$represents the photocurrent, $I_{\text{0}}$ is the current flowing through the diode, $I_{sh}$ is the current of the shunt resistor, $R_{sh}$ is the parallel resistor, $R_{s}$ is the series resistor, U is the output voltage of the photovoltaic cell, and I is the output current of the photovoltaic cell [3].

From the equivalent model in Fig 2, the mathematical model formula of the photovoltaic cell is derived as


$$I=I_{ph}-I_{\text{0}}-I_{sh}$$ (3)

Block Parameters In the PV photovoltaic panel, the specifications are as shown in Table 2.

**Table 2:** Specifications of Photovoltaic Panels.

Parameter	Value
Open circuit voltage Voc (V)	36.3 V
Voltage at maximum power point Vmp (V)	29 V
Short-circuit current Isc (A)	7.84 A
Current at maximum power point Imp (A)	7.35 A
Cells per module (Ncell)	60
Parallel strings	2
Series-connected modules per string	1
Maximum Power (W)	213.15 W

**Fig 3:**
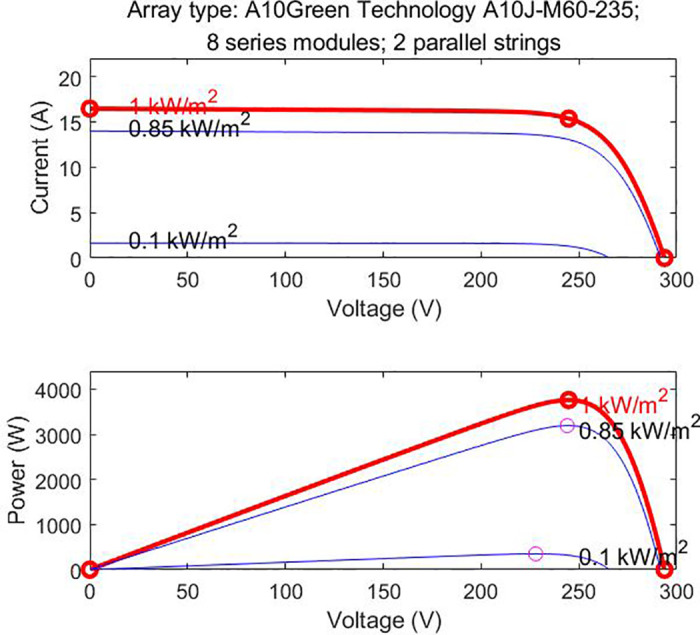
The I-U/P-U characteristic curve of the photovoltaic module.

Light intensity is another important external factor affecting the output characteristics of photovoltaic modules. Fig 3 shows the I-U and P-U output characteristic curves of the photovoltaic module at 25°C temperature under different light intensities (1000W/㎡, 500W/㎡, 100W/㎡). The most obvious change is that for every increase of 500W/㎡ in light intensity, the short-circuit current of the module increases by approximately 7A and the power increases by about 500W.

Under the local shading condition, the output of the photovoltaic module presents a multi-peak characteristic curve. The influence of shading on the output characteristics is quite obvious. When the shading rate k = 0.1, 10% of the light is blocked, and 1 – k represents 90% of the light on the photovoltaic array. The photocurrent can be expressed as


$$I_{ph}=\frac{(\text{1}-k)G}{\text{1}\text{0}\text{0}\text{0}}I_{ph\text{0}}$$ (4)

**Fig 4:**
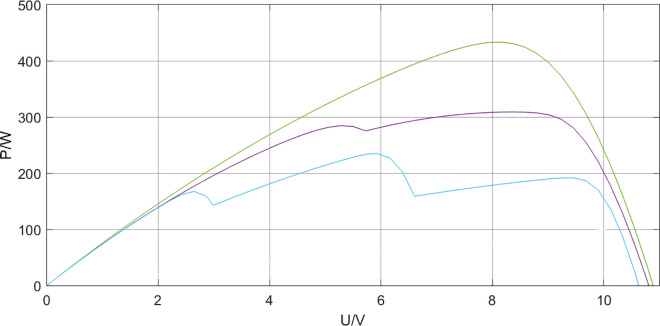
Solar P-V output characteristic curves under normal lighting conditions and partial shading conditions.

**Fig 5:**
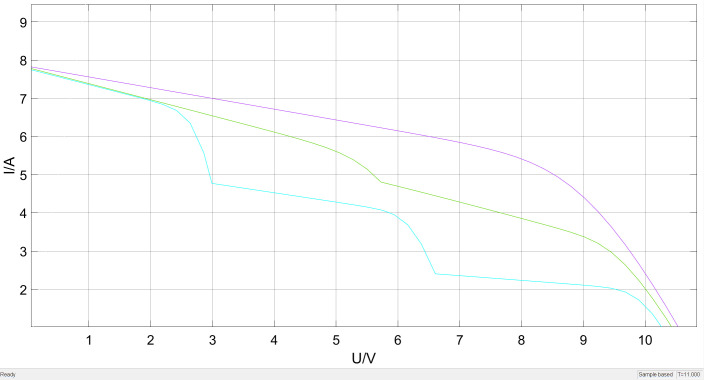
Solar I-V output characteristic curves under normal lighting conditions and partial shading conditions.

In the formula, ${\text{I}}_{\text{ph0}}$ represents the photocurrent under standard test conditions; G represents the solar irradiance intensity before the shading object. When the photovoltaic cell is in a shadow state under complex light conditions of the photovoltaic module, the output efficiency will decrease, and the voltage and power will present a multi-peak characteristic curve.This paper investigates the multi-peak state of the P-U and I-U output curves of photovoltaic modules at a temperature of 25°C. Under different light distribution scenarios, the light intensities in each area are 500, 600, and 1000 W/㎡. The power-voltage (P-U) and Current-Voltage (I-U) characteristic curves of the photovoltaic array all exhibit the phenomenon of triple peaks. This is because the number of peaks is related to the illumination level. After reaching the first peak, the single-peak curve still rises, so before reaching the peak point, it immediately enters the next peak. The specific P-U and I-U output curves of the photovoltaic array are shown in Fig 4 and 5.

## The strategy of the refined hierarchical population algorithm (PSSSA)

### PSO algorithm

The traditional PSO algorithm originated from the observation of bird flocks. This algorithm has a simple structure, fast convergence speed, high optimization accuracy, and is suitable for finding the extremum in nonlinear problems. In the N-dimensional solution space, after a group consisting of m particles undergoes a round of iteration, the spatial coordinates of the i-th particle can be expressed as ${X_{i}^{a}=(X}_{i\text{1}}^{a},X_{i\text{2}}^{a},X_{i\text{3}}^{a},X_{i\text{4}}^{a},...,X_{iN}^{a}$*)*, after a round of iteration, the individual optimal solution of the i-th particle is represented by vector $P_{best}^{a}=(P_{best,\text{1}}^{a},P_{best,\text{2}}^{a},...,P_{best,N}^{a}$), the global optimal solution vector of the particle swarm is represented by vector $P_{g,best}^{a}=(P_{g,best,\text{1}}^{a},P_{g,best,\text{2}}^{a},...,P_{g,best,N}^{a})$, nd the velocity of the i-th particle after a round of iteration is represented by vector $V_{i}^{a}=(V_{i\text{1}}^{a},V_{i\text{2}}^{a},V_{i\text{3}}^{a},...,V_{iN}^{a})$, Thus, the mathematical expression of the particle swarm can be obtained as [8]


$$v_{in}^{a+\text{1}}=\omega v_{in}^{a}+c_{\text{1}}r_{\text{1}}\left(p_{best,in}^{a}-x_{in}^{a}\right)+c_{\text{2}}r_{\text{2}}\left(p_{g,best}^{a}-x_{in}^{a}\right)$$ (5)

$$x_{in}^{a+\text{1}}=x_{in}^{a}+v_{in}^{a+\text{1}}$$ (6)

In this formula, ω represents the inertial weight of the particle’s motion; $c_{\text{1}}$ is the self-influence factor of the particle; $c_{\text{2}}$ is the interaction factor between particles; $r_{\text{1}},\;r_{\text{2}}$ is a constant ranging from 0 to 1. However, it is prone to getting trapped in local optima, and its ability to handle high-dimensional complex problems decreases. The diversity of the population may rapidly diminish. Moreover, the SSA algorithm performs well in local search and can quickly converge to the vicinity of the optimal solution. However, the global search ability of SSA is relatively weak, and it may get trapped in local optima in complex multi-peaked problems.

### Detailed hierarchical population algorithm (PSSSA) strategy

**Fig 6:**
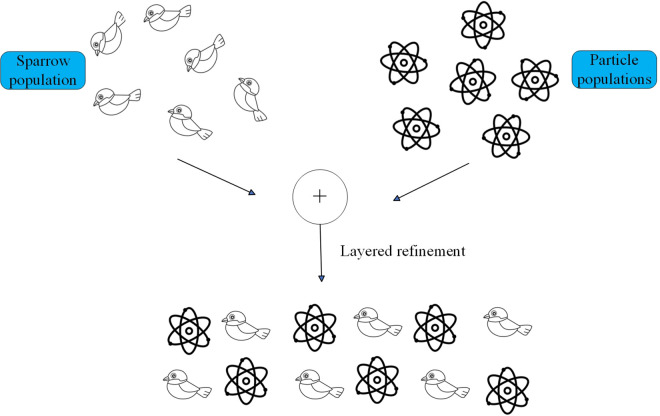
Hierarchical refinement of the model diagram.

This paper proposes an improved MPPT control strategy based on the refined hierarchical population algorithm. This strategy introduces a PSO population pause mechanism, aiming to avoid the premature convergence problem of PSO. Secondly, a refined hierarchical sequence is applied to the individuals within the SSA and PSO populations to generate an evenly distributed initial population, and the finch and particle populations are designed to collaboratively search for the optimal solution, enhancing the algorithm’s compatibility. The two populations are decomposed to optimize the number of individuals in the population, update the optimal fitness value of the population individuals; and the population positions are reorganized in each layer, enabling each layer to independently optimize, making the entire population easier to collaborate and improving population diversity, thereby increasing search efficiency. Finally, the Padding function and symbol matrix strategies are introduced in the velocity update position of PSSSA to reduce the adverse effects of boundary effects on power fluctuations, effectively improving tracking errors and power loss. The specific refined hierarchical principle model diagram is shown in Fig 6. In the left side of Fig 6, there is the SSA population, on the right side is the PSO population, and at the bottom is the population size after refined hierarchical processing.

### Refine the hierarchical initialization of the population

Firstly, the PSO population suspension mechanism is introduced. The collaboration of the sparrow and particle populations for optimization is designed. A refined hierarchical sequence is adopted to generate an evenly distributed initial population. A part of the sparrow population is refined and incorporated into the particle population. The discovery, danger, and joining roles of the sparrow population are identified within the particle population, and the collaboration of the sparrow and particle populations for optimization is designed.

The producer position formula is added successively:


$$x_{i}^{t+\text{1}}=\left\{ \begin{array}{c} x_{i}^{t}exp\left(\frac{-i}{\beta T_{max}}\right),q<st,\\ x_{i}^{t}+RL,\;q\geq t, \end{array}\right.\;$$ (7)

In the formula: i = 1, 2,..., n, t represents the number of iterations, R is a random number following a normal distribution, L is a 1 × d matrix with each element being 1, q (warning value) ∈ [0, 1], st (safety value) ∈ [0.5, 1].

When the producer senses danger, some sparrows will follow the producer and act together. However, once the producer finds a food source, these followers will attempt to seize the food. In this case, a portion of the sparrows may fly to other areas to search for food to meet their energy requirements. The position update formula is the formula for the position of the joiner:


$$x_{i}^{t+\text{1}}=\left\{ \begin{array}{c} Rexp\left(\frac{G_{worst}-x_{i}^{t}}{i^{\text{2}}}\right),i>\frac{n}{\text{2}},\\ S_{best}+\left|x_{i}^{t}-S_{best}\right|C^{+}L,i\leq \frac{n}{\text{2}}, \end{array}\right.\;$$ (8)

In the formula: $G_{worst}$ represents the worst position of the sparrow, $S_{best}$ represents the best position of the producer, *C* is a (1 × d) random vector, with element values defined as 1 or −1, $C^{+}=C^{T}{\left(CC^{T}\right)}^{-\text{1}}$, and n is the number of the population.

During the foraging activities of the sparrows, some members of the group play the role of discoverers. Their main responsibility is to monitor potential threats in the environment. When these discoverers detect the presence of predators, they will quickly interrupt the foraging action to ensure the safety of the group, and by specific behaviors or sound signals guide other sparrows to quickly move to a safe area. The position update formula is the danger position formula:


$$x_{i}^{t+\text{1}}=\left\{ \begin{array}{c} @lG_{best}+T\left|x_{i}^{t}-G_{best}\right|,f\left(x_{i}^{t}\right)>f\left(G_{best}\right)\\ x_{i}^{t}+S\frac{\left|x_{i}^{t}-G_{worst}\right|}{f\left(x_{i}^{t}\right)-f\left(G_{worst}\right)+\delta },f\left(x_{i}^{t}\right)=f\left(G_{bestt}\right) \end{array}\right.\;$$ (9)

In the formula: $G_{best}$ represents the current global optimal position, T is a random number following a normal distribution, $\text{f}\left(x_{i}^{t}\right)$ is the sparrow’s fitness, $\text{f}\left(G_{worst}\right)$ is the global best fitness, S is a random number in the range of [−1, 1], and δ is the minimum constant.

Based on the advantages of SSA and PSO, the sparrow and particle groups are designed to collaborate in optimizing, enhancing the diversity of the population; the two major populations are decomposed to optimize the number of individuals in the group, update the optimal fitness value of the individual in the population; and the population positions are reorganized at each layer, so that each layer can independently optimize. A refined hierarchical sequence is used to generate an evenly distributed initial PSSSA population, and the initial parameters of the algorithm are set, including the size of the population n, the maximum number of iterations Tmax, and the dimension of the search space n. The initial positions of the individuals in each population are as follows:


$$X=[\begin{array}{ccc} x_{\text{1},\text{1}} & \cdots & x_{\text{1},n}\\ \vdots & \ddots & \vdots \\ x_{n,\text{1}} & \cdots & x_{n,n} \end{array}]$$ (10)

Based on this, in order to further suppress the premature convergence phenomenon of the PSO algorithm, by dividing and layering the discoverer, joiner and danger node in the sparrow algorithm into the velocity equation of the PSO algorithm, the improved velocity equation is expressed as:


$$v_{n}=\omega v_{n}+\left(c_{\text{1}}r_{\text{1}}\left(p_{id}-(x_{n}+p_{f}+p_{j}+p_{d})\right)\right)+\left(c_{\text{2}}r_{\text{1}}\left(p_{gd}-(x_{n}+p_{f}+p_{j}+p_{d})\right)\right)$$ (11)

In the formula: ω represents the weight coefficient; $v_{n}$ represents the speed; $c_{\text{1}},\;c_{\text{2}}$ represents the learning factor, with initial values of 1.2 and 2 respectively; $p_{id}$ represents the individual optimal position of the particle swarm; $p_{f}$ represents the producer position of the sparrow population; $p_{j}$represents the joiner position of the sparrow population; $p_{d}$ represents the dangerous position of the sparrow population; $p_{gd}$ represents the global optimal position of the particle swarm; $r_{\text{1}}$ is a constant. To further verify the improvement effect, this paper conducts a comparative analysis on the situation where only a single parameter is added. However, the experimental results show that the initial power fluctuation and convergence effect have not been significantly improved. Therefore, in the following text, the algorithm strategy will be further optimized by introducing strategies such as the Padding function and the symbol matrix, in order to further enhance the algorithm performance.

### Construction of chaos coefficient and boundary processing function

In order to achieve the search accuracy and convergence speed of the PSSSA algorithm and reduce the influence of power fluctuations during the tracking process. Finally, chaotic coefficients, Padding functions, symbol matrices, etc. are introduced at the position of speed update. The chaotic (a concept in nonlinear science) coefficient has a strong measurement standard for random motion. At the same time, in order to improve the stability and controllability of the algorithm and reduce the chaotic degree of the algorithm, the Tent (chaotic coefficient) is added. The formula is as follows:


$$Y_{k+\text{1}}=\left\{ \begin{array}{c} @r\frac{Y_{k}}{\beta },\;Y_{k}\in (\text{0},\beta ]\\ \frac{\text{1}-Y_{k}}{\text{1}-\beta },Y_{k}\in (\beta ,\text{1}] \end{array}\right.\;$$ (12)

In the formula: β is the constant in the chaos coefficient, $Y_{k}$ is the size of the chaos coefficient at time k.

When the system detects that the deviation between the real-time power value and the target power value exceeds the preset threshold (that is, the power difference is less than 5% of the target power value), the power correction mechanism will be automatically triggered. By executing the restart algorithm, the system power output will be recalibrated to ensure that the power value is stable within the allowable error range, achieving precise power control.

This is to solve the problems of stagnation in the optimization process of PSO, large search range, and large power fluctuations. From formula (13), it can be seen that the new velocity update formula is related to the parameters of the sparrow population and the parameters of the PSO operating conditions (such as the individual optimal position).


$$v_{n}=\omega v_{n}+\left(c_{\text{1}}Y_{k+\text{1}}\left(p_{id}-x_{n}\right)\right)+\left(c_{\text{2}}Y_{k+\text{1}}\left(p_{gd}-x_{n}\right)\right)$$ (13)

**Fig 7:**
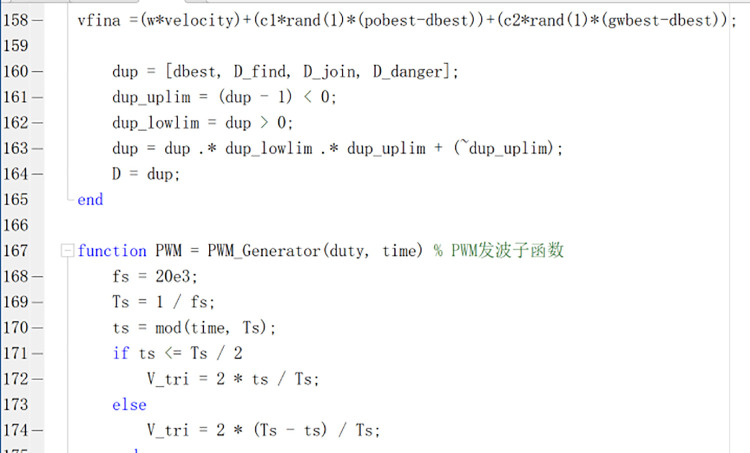
Optimized Code Diagram.

**Fig 8:**
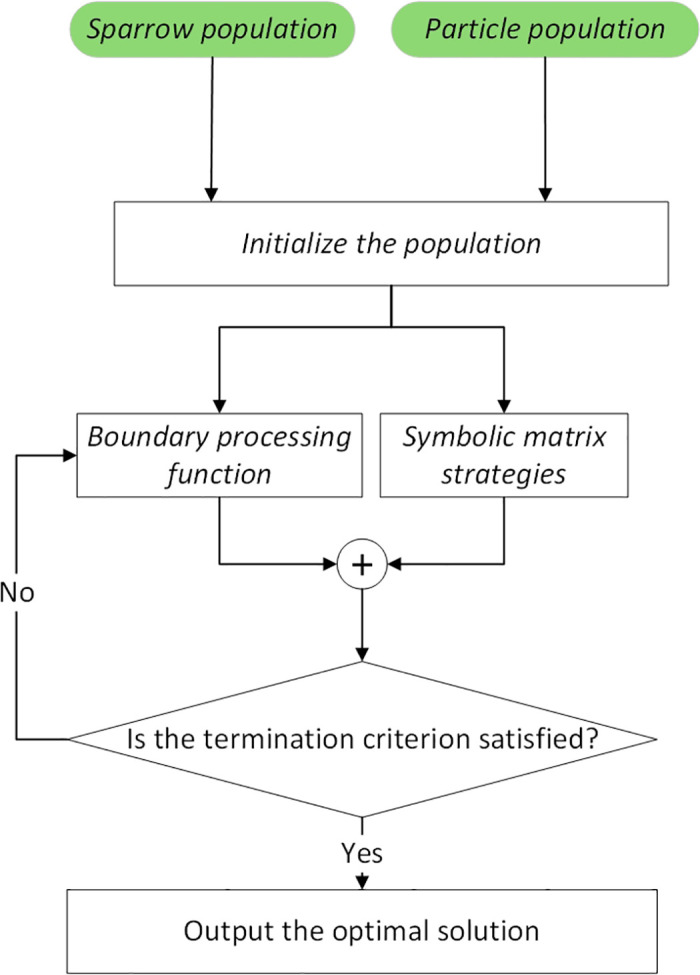
Flowchart of the Principle of the Detailed Hierarchical Population Algorithm.


In the formula: ω is the weight coefficient; $v_{n}$ is the speed; $x_{n}$ is the particle position; $c_{\text{1}},\;c_{\text{2}}$ is the learning factor, with initial values of 1.2 and 2 respectively; $p_{id}$ is the optimal position of the individual in the PSSSA population; $p_{gd}$ is the global optimal position of the PSSSA population; ${\text{Y}}_{\text{k}+\text{1}}$ is the Tent (chaotic coefficient). Fig 7 shows a part of the code after hierarchical refinement of the population. When using the PSSSA algorithm for multi-model experiments, the experimental parameters must be at the same order of magnitude. In this model, simulation and laboratory analysis were conducted respectively. Good results were achieved in the experiments, and the power curve and parameter values were improved. This indicates that the PSSSA algorithm can effectively handle complex data. Fig 8 is the schematic diagram of the PSSSA algorithm.

$$f(x)=V_{pv}\cdot I_{pv}$$ (14)

Formula 14 represents the algorithm optimization objective function. $V_{pv}$ and $I_{pv}$ are the output voltage and current of the photovoltaic array obtained through real-time sampling. This paper focuses on the maximum power point tracking (MPPT) problem of photovoltaic systems under uniform and non-uniform light conditions. This problem is essentially a dynamic optimization problem that needs to be solved. In the algorithm design, we take the duty cycle D of the photovoltaic DC-DC converter as the decision variable, and the position vector of each individual in the algorithm represents a candidate duty cycle value. The optimization objective is to maximize the output power of the photovoltaic array in real time. To this end, we directly define the objective function as the output power calculated by formula 14. Thus, the engineering optimization problem of MPPT is directly transformed into a function optimization problem that the algorithm can handle. Under this model, each fitness evaluation of the algorithm corresponds to one power sampling of the actual system, ensuring that the optimization goal of the algorithm is completely consistent with the physical goal of MPPT, and achieving a closed-loop mapping from algorithm iteration to system power response. In the experimental part of Chapter 4 of this paper, when the proposed algorithm is applied to MPPT, this power function is used as the sole objective function for evaluation.

**Fig 9:**
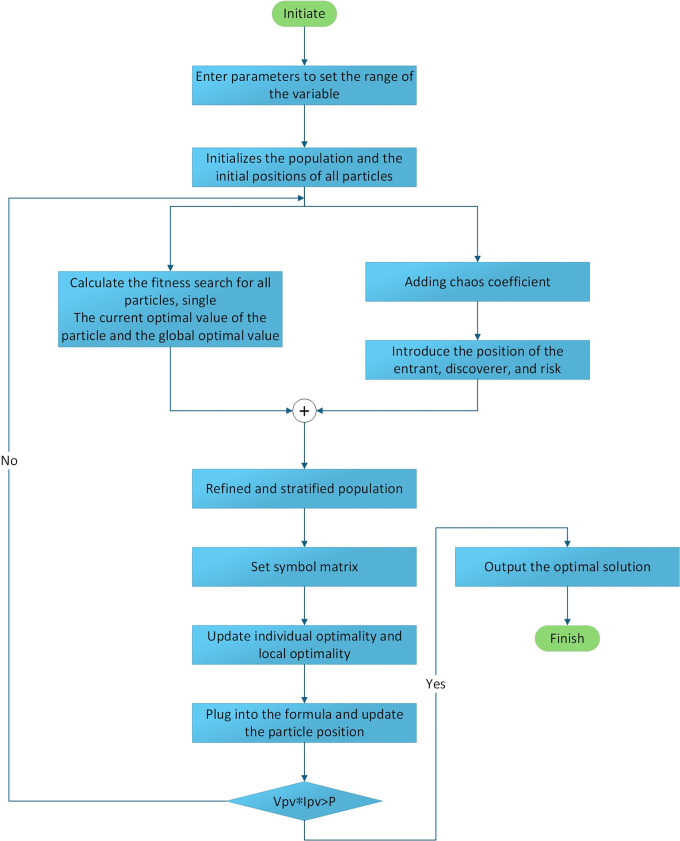
Flowchart of the refined hierarchical population algorithm.

This integration strategy can effectively prevent premature convergence to local optimal solutions during the initial iteration stage. In the optimization process of the PSO population, a PSO population pause mechanism is introduced. By using Equation (10), a refined hierarchical sequence is applied to the current SSA and PSO populations to generate an evenly distributed initial population. The fitness values of the stratified populations after the update are recalculated. The collaboration of the sparrow and particle populations for optimization is designed, and the optimal fitness values of each of the two populations are selected and stored. Then, the positions of the individuals within the population are reorganized. When the algorithm determines that it has fallen into an early convergence state, the positions and velocities of the population individuals are updated. According to Equation (13), strategies such as the Padding function and symbol matrix are introduced for local search. Taking the optimal position of the PSSSA population individual $p_{id}$ and the global optimal position$p_{gd}$ of the PSSSA population as a reference value, the search range is narrowed, and a pattern search is conducted centered on the reference value. The fitness values of the individuals within each population are compared with the previous ones to find the optimal fitness value of the population. Finally, the position update mechanism is used to ensure that the current solution is superior to the historical optimal solution. This algorithm has been successfully implemented and verified in the Simulink simulation platform. The flowchart of the integration algorithm is as follows: shown in Fig 9. The algorithm flow depicted in Fig 9 can be systematically mapped to the three stages of the standard MPPT process: startup initialization, dynamic tracking and optimization, and convergence and output. Firstly, in the startup and initialization stage, the algorithm uses chaotic mapping to generate an initial population covering the entire voltage feasible region and completes the first power sampling, effectively avoiding the risk of traditional methods falling into local optimum due to improper initial voltage setting. Secondly, in the dynamic tracking and optimization stage, in the core loop of the algorithm, the system executes intelligent disturbance, power evaluation, and individual position update in sequence within each control cycle, driving the operating voltage to gradually approach the global maximum power point. During this process, the Padding function adaptsively optimizes the disturbance behavior to enhance search stability, while the boundary handling mechanism ensures that the generated voltage command always remains within the feasible range. Finally, when the preset convergence conditions are met, the algorithm enters the convergence and output stage, locking in the optimal voltage command and maintaining the photovoltaic system in operation at the maximum power point. This framework has the ability to autonomously restart, and if it detects a power drop caused by environmental changes, it can trigger the above process again to achieve continuous and adaptive maximum power point tracking.

## Simulation results and analysis

**Fig 10:**
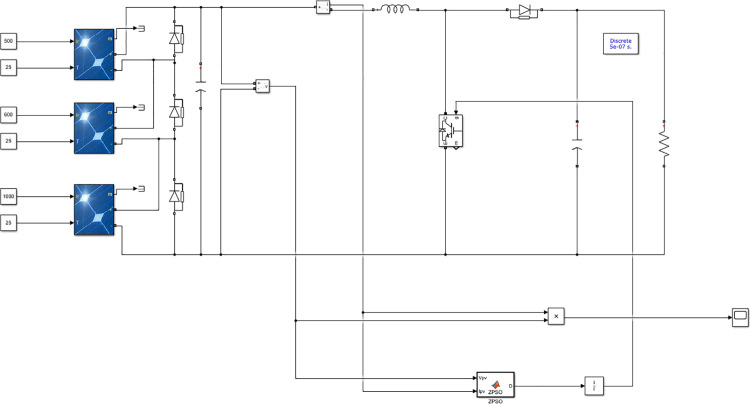
Simulation Model Diagram.

In MATLAB/Simulink, a complex lighting model was established. The Block Parameters PV photovoltaic panel circuit layout was used, and the Boost boost circuit was adopted to build the model diagram under uniform lighting conditions. The PWM was converted into a duty cycle signal to control the switch, thereby forming an equivalent load between the Boost boost circuit and the load, achieving the control of MPPT time. The simulation model diagram is shown in Fig 10. Based on the light analysis of the Northeast region and combined with the basic situation diagram of the laboratory 11, this experiment was conducted under both uniform lighting and complex lighting conditions. The open-circuit voltage ${\text{U}}_{\text{oc}}$ of the photovoltaic cell model was set at 36.3 V, the short-circuit current $I_{SC}$ at 7.84 A, the peak voltage$U_{m}$at 29 V, and the peak current $I_{m}$ at 7.35 A. The MPPT module, boost inductor, PWM converter, filter capacitor, load, and the photovoltaic cell temperature set at 25 °C and the illumination at 1000 W/㎡ were all included under the uniform lighting condition. Table 3 shows the parameters of each component. During the experiment, no specific fine-tuning of any algorithm was conducted based on any particular test function. For the PSSSA algorithm, its unique parameters were tested using a set of fixed values across all test problems to ensure fairness when comparing the MPPT algorithms. Each experiment under each light intensity condition is independently repeated 3 times. Data collection and analysis are completed through a custom MATLAB script, and the results are expressed as the average value ± standard deviation. The specific experimental results are as follows. The summary table of algorithm parameter settings is shown in Table 4.

**Table 3:** Test Component Parameters.

Parameter	Value
Series L Branch	1e-3 H
Series R Branch	1 ohms
Series C Branch	300e-6 F
PWM	5000 Hz
Diode	0.8 V

**Table 4:** Comparison Table of Algorithm Parameter Settings.

Title	Public parameters	Parameter Values and Basis Explanation
PSO	N = 30, Max_iter = 1000	Using the standard parameters set for PSO, which are widely adopted in the literature, ensures the benchmark nature of the comparison.
SSA	N = 30, Max_iter = 1000	Use the default parameters recommended in the original SSA paper
PSSSA	N = 30, Max_iter = 1000	The parameters of PSSSA were all optimized and determined within their reasonable ranges through pre-experiments, and the number and complexity of its parameters were not significantly higher than those of other comparison algorithms.
ISSA	N = 30, Max_iter = 1000	Use the default parameters recommended in the original ISSA paper

**Fig 11:**
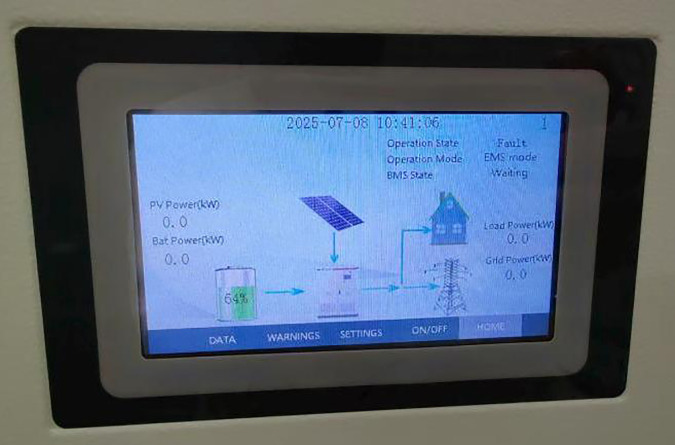
Laboratory System.

To verify the effectiveness of the fusion algorithm, in this simulation experiment, it was simulated and analyzed under two conditions: uniform illumination and complex illumination. The MPPT simulation was conducted successively for the traditional PSO algorithm, chaotic PSO algorithm, PSSSA algorithm, sparrow algorithm, and perturbation observation method. Fig 16 shows the experimental operation model diagram Fig 11.

### Uniform illumination simulation analysis

Table 5 shows the time required to reach the maximum power under uniform illumination conditions.

**Table 5:** Results of Uniform Illumination Test.

Method	Evaluated parameter	
	Nature of tracking waveforms	Power tracking(W)
PSSSA	Less oscillatory and stable	310.9 W
ISSA	Oscillatory	290.6 W
SSA	Oscillatory	270.5 W
PSO	Oscillatory	280.1 W

**Fig 12:**
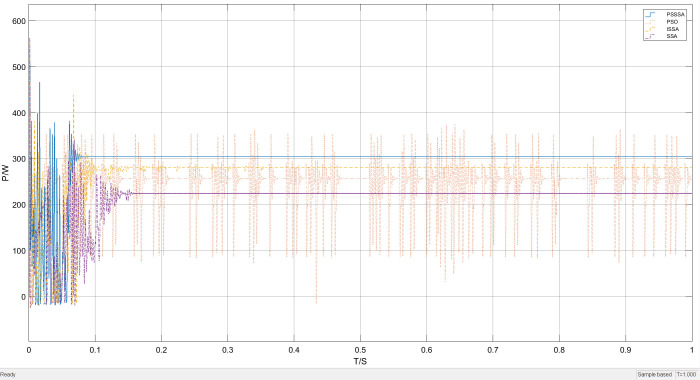
Uniform illumination power tracking curve graph.

**Fig 13:**
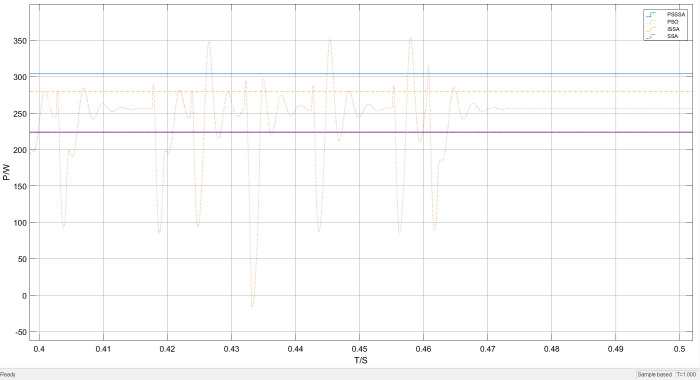
Uniformly illuminated part enlarged power tracking curve graph.


Under unobstructed conditions, the overall waveform of power tracking is shown in Fig 12. It can be clearly seen from Fig 12 that the time required to track the maximum power point varies among the four methods under unobstructed conditions. As shown in the local magnification of Fig 13, the time required for SSA-MPPT and ISSA-MPPT to reach the maximum power point is 0.14 seconds and 0.11 seconds respectively, with the maximum power being 270 watts and 290 watts respectively. However, PSO-MPPT fluctuates after stabilizing for a period of time and eventually stabilizes at 280 watts. Compared with PSSSA-MPPT, the tracking time is approximately 0.08 seconds and the maximum tracked power is 310 watts. Under uniform illumination, the improved algorithm has a smoother tracking waveform, a smaller steady-state oscillation rate, and a more stable tracking power effect compared to the traditional PSO. Compared with the improved sparrow algorithm, the improved sparrow algorithm finds the maximum power point after 0.11 seconds, but there is a large power fluctuation near the maximum power point. This is because in the process of sparrows foraging for food, some members of the group play the role of discoverers, whose main responsibility is to monitor potential threats in the environment. When these discoverers detect the presence of danger, they will quickly interrupt the foraging activity to ensure the safety of the group. And guide other sparrows to quickly move to a safe area through specific behaviors or sound signals, which leads to a decrease in the overall tracking efficiency of the algorithm. In contrast, PSO-MPPT, SSA-MPPT, and ISSA-MPPT increase the power by 40 watts, 30 watts, and 20 watts respectively. The corresponding maximum power growth rates are A = 14.81%, B = 10.71%, and C = 6.7%, and the calculation method of the maximum power increase rate is as follows:

$$A=\frac{P_{PSSSA}-P_{PSO}}{P_{PSSSA}}$$ (15)

$$B=\frac{P_{PSSSA}-P_{SSA}}{P_{PSSSA}}$$ (16)

$$C=\frac{P_{PSSSA}-P_{ISSA}}{P_{PSSSA}}$$ (17)

In the formula: $P_{PSSSA},\;P_{PSO}$、$P_{SSA}$ and $P_{ISSA}$ are the maximum power values of PSSSA, PSO, SSA, and ISSA respectively. As can be seen from Fig 12 to 13, compared with the traditional PSO method, the improved PSSSA algorithm demonstrates more stable tracking characteristics and has lower steady-state fluctuations. This is because the sparrows and the particle swarm cooperate in optimizing, thereby enhancing the diversity of the population. The PSSSA algorithm can complete the maximum power point tracking within 0.08 seconds, and the tracking power is more stable. In contrast, the traditional particle swarm optimization algorithm has a more obvious waveform oscillation when locating the maximum power point, and significant power oscillations occur when approaching the maximum power point, resulting in an output power of 280 W. This phenomenon is due to the relatively scattered particle distribution in the initial stage, causing individuals to have difficulty responding quickly to environmental changes and adjusting the search direction; while in the later stage of the search, some particles turn to explore other areas, reducing the overall tracking efficiency. Although the power is relatively large, the output is unstable, thereby affecting the continuous output of photovoltaic power generation.

The improved PSSSA algorithm demonstrates significantly better tracking stability and power output compared to the comparison algorithm. The optimal output power shows the highest tracking accuracy with a stable performance. This performance improvement is attributed to the introduction of power restart mechanism and Padding function, as well as strategies such as symbol matrix in the later stages of the algorithm. By directly adjusting to locate the maximum power point and combining with power abnormal restart strategy, it effectively avoids the high-frequency oscillation problem caused by multiple iterations, while enhancing the search accuracy. This proves that the integrated algorithm has better dynamic response capability in uniform illumination. Since uniform illumination was used in this experiment and the conditions such as light intensity remained unchanged, the experimental results are insufficient to prove the effectiveness of this algorithm. Therefore, the tracking effect simulation under the MPPT algorithm under the distribution of sudden light changes is shown as follows.

### Simulation analysis of light mutation

**Fig 14:**
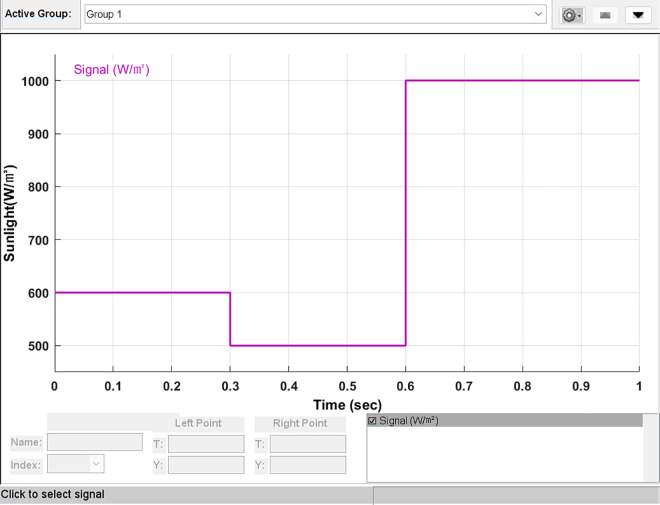
Sun radiation intensity variation graph.

To verify the performance of the proposed PSSSA algorithm, irradiation with both ascending and descending shapes was used. The irradiance range was between 500 W/m^2^ and 1000 W/m^2^. The lighting distribution is shown in Fig 14. The irradiance started at 600 W/m2 and remained at this level for 0.3 seconds; then it decreased to 500 W/m^2^ and remained at this level for 0.3 seconds; subsequently, it increased to 1000 W/㎡ and remained flat for 0.4 seconds. Since we are focusing on the steady-state conditions in the algorithm, the ascending or descending processes in the irradiance distribution were not considered. The simulation time was 1 second, and the temperature was fixed at 25 °C.

This determined the tracking efficiency of each MPPT. After simulation studies, the test results showing the performance under sudden changes in irradiance levels were presented in Table 6.

**Table 6:** The test results of sudden changes in irradiance levels.

Method	Evaluated parameter	
	Nature of tracking waveforms	Power tracking(W)
PSSSA	Less oscillatory and stable	330 W
ISSA	Less oscillatory and stable	310 W
SSA	Oscillatory	290 W
PSO	Oscillatory	270 W

**Fig 15:**
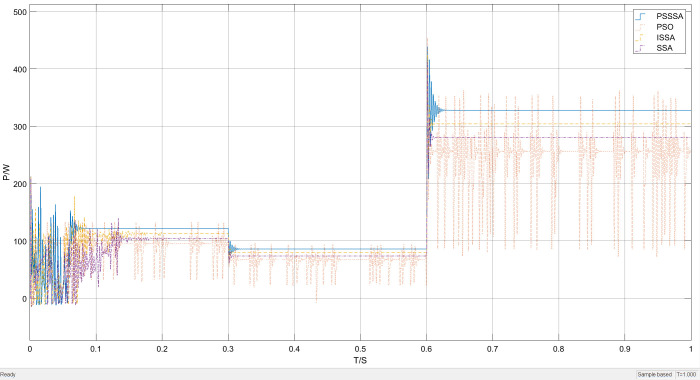
Lighting mutation power tracking curve graph.

Fig 15 shows the overall waveform performance comparison of power tracking under the condition of light intensity variation. It can be clearly seen from Fig 15 that the tracking effect of the PSSSA algorithm is greater. This is because the addition of the power restart mechanism effectively avoids the problem of high-frequency oscillation caused by multiple iterations, reduces system energy loss, and simultaneously improves search accuracy.

**Fig 16:**
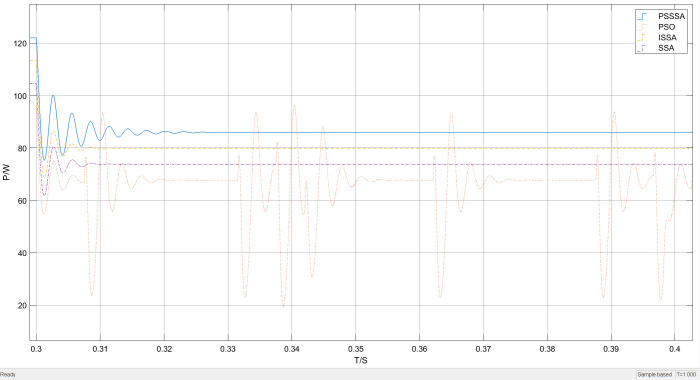
Lighting mutation part enlarged power tracking curve graph.

By using local magnification, the performance of this MPPT algorithm under different irradiance levels was emphasized. The results show that the proposed algorithm has better stability. Fig 16 presents a comparison of the local magnification of the light intensity sudden change tracking. The former performs better in reducing oscillations, but in terms of tracking performance, the proposed algorithm has a better tracking effect.

### Simulation analysis of temperature sudden change

**Fig 17:**
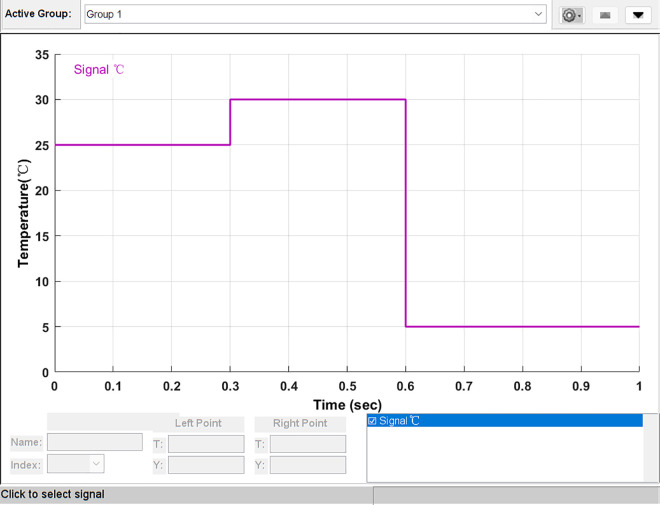
Sun radiation intensity variation graph.

To verify the performance of the proposed PSSSA algorithm, an ascending and descending temperature pattern was adopted. The temperature variation range was between 30°C and 5°C. The temperature distribution is shown in Fig 17. The temperature started at 25°C and remained at this level for 0.3 seconds; then it increased to 30°C and remained at this level for 0.3 seconds; subsequently, it decreased to 5°C and remained stable for 0.4 seconds. Since we focused on the steady-state conditions in the algorithm, the ascending or descending processes in the temperature distribution were not considered. The simulation time was 1 second, and the light intensity was fixed at 1000 W/m^2^.

This determines the tracking efficiency of each MPPT. Through simulation studies, the performance test results under sudden temperature changes are presented in Table 7.

**Table 7:** The test results of sudden changes in temperature levels.

Method	Evaluated parameter	
	Nature of tracking waveforms	Power tracking(W)
PSSSA	Less oscillatory and stable	330 W
ISSA	Less oscillatory and stable	290 W
SSA	Oscillatory	270 W
PSO	Oscillatory	230 W

**Fig 18:**
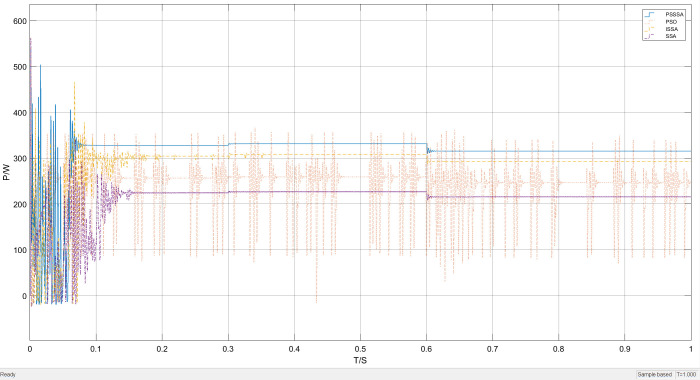
Lighting mutation power tracking curve graph.

Fig 18 presents the overall waveform performance comparison of power tracking under temperature variation conditions. It can be clearly seen from Fig 18 that the tracking effect of the PSSSA algorithm is better. This is because the population undergoes a refined hierarchical sequence, thereby achieving a collaborative optimization mechanism that effectively avoids the high-frequency oscillation problem caused by multiple iterations. This not only effectively reduces the overall system energy consumption but also simultaneously achieves a synchronous improvement in search accuracy.

**Fig 19:**
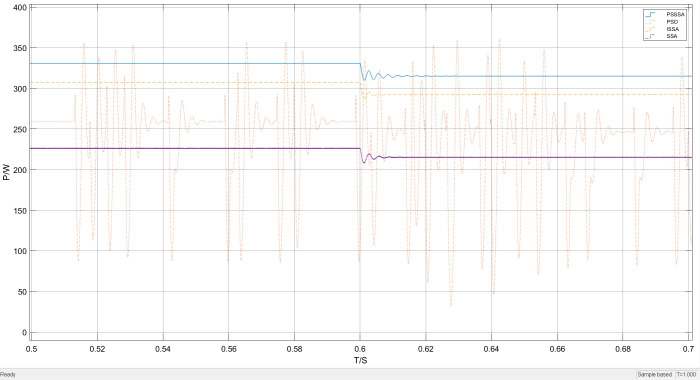
Lighting mutation part enlarged power tracking curve graph.

By employing the local magnification technique, the performance of this MPPT algorithm has been significantly demonstrated under different temperature levels. The results show that the proposed algorithm has higher stability. Fig 19 presents the comparison of tracking local magnification under sudden temperature changes. The experimental results indicate that the performance of the algorithm is complementary under dynamic conditions. Although the former has smaller output fluctuations in the steady state, the proposed algorithm achieves a better balance in convergence accuracy and tracking speed, demonstrating stronger global optimization ability.

### Complex lighting simulation analysis

In this study, since the light intensity in the actual environment is subject to dynamic changes, a novel PSSSA was proposed to solve the global optimization problem in complex lighting conditions. The light intensity in complex lighting conditions was set at 500, 600, and 1000 W/㎡. In PSSSA, the sparrow population and the particle population were combined to generate a high-quality initial population. Then, the refined hierarchical population strategy was applied to balance exploration and exploitation. Finally, a hybrid perturbation mechanism was embedded in PSO to increase the probability of escaping from local optima. Compared with SSA and other advanced algorithms, PSSSA has advantages in solution accuracy, convergence speed, scalability, and stability. Moreover, the proposed PSSSA was applied to solve two real-world optimization problems. The experimental results show that PSSSA is a practical and effective method for handling complex engineering optimization problems. Simulation comparisons of the maximum power point tracking (MPPT) performance of the three algorithms were conducted under sudden and uniform lighting conditions. The results are shown in Table 8.

**Table 8:** Shadow Illumination Test Results Table.

Method	Evaluated parameter	
	Nature of tracking waveforms	Power tracking(W)
PSSSA	Less oscillatory and stable	290 W
ISSA	Less oscillatory and stable	220 W
SSA	Oscillatory	180 W
P&O	Oscillatory	100 W

**Fig 20:**
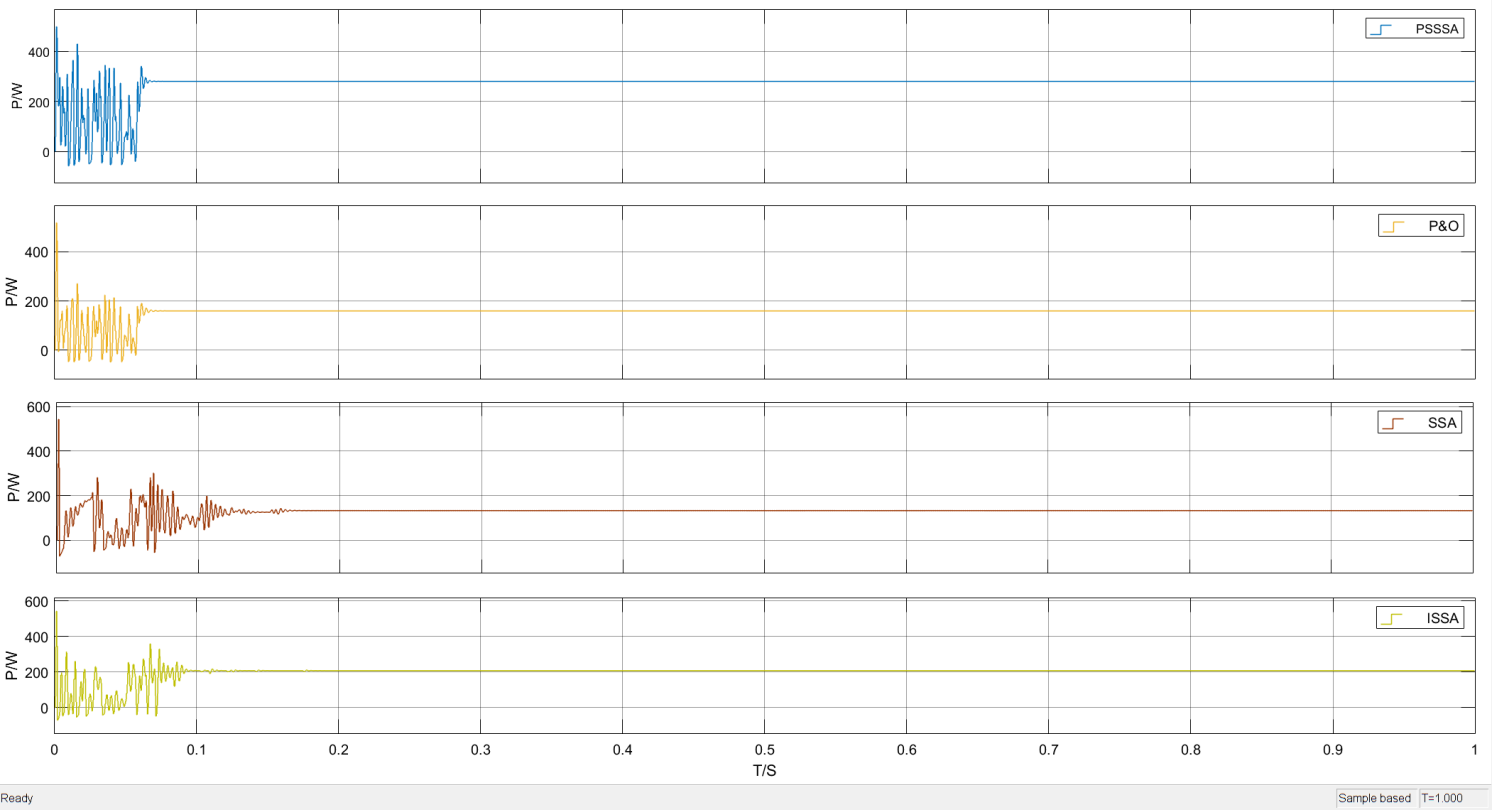
Shadow condition power tracking curve graph.

By comparing the maximum power point tracking (MPPT) simulation results of the three algorithms under shadow conditions, Fig 20 shows that in an environment with shadow distribution, the improved PSSSA algorithm, compared with the traditional SSA method, not only shows a more stable tracking waveform in terms of power size and tracking speed, but also has a lower steady-state oscillation rate. Moreover, it can complete the tracking in less than 0.1 seconds, with an output power of approximately 290 W. In contrast, the traditional SSA method takes 0.15 seconds to locate the maximum power point, and exhibits significant power fluctuations near the maximum power point, with an output power of approximately 180 W. The improved SSA algorithm also reaches the maximum power within 0.15 seconds, with an output power of approximately 220 W. This difference mainly stems from the relatively scattered population distribution in the initial stage of the algorithm, which makes it difficult for individuals to quickly respond to environmental changes and adjust the search direction; while in the later stage of the search, some sparrows turn to explore other areas, further reducing the overall tracking efficiency.

The improved PSSSA algorithm can accurately track the maximum power point in approximately 0.1 seconds. Its tracking stability is significantly better than the other three comparison algorithms. The optimal output power reaches 290 W, demonstrating the highest tracking accuracy. This performance improvement is mainly attributed to the introduction of power restart mechanism, Padding function, symbol matrix and other strategies in the later stage of the algorithm, which effectively avoids the high-frequency oscillation problem caused by multiple iterations, reduces system energy loss, and improves search accuracy. Considering that the light intensity in the actual environment is always in a dynamic changing state, it is necessary to compare the tracking effect of the MPPT algorithm under dynamic shadow distribution. During the simulation process, the environmental conditions of the photovoltaic array changed from uniform light distribution to shadow distribution. As shown in Fig 20, when the external environment was in shadow distribution, the power of this algorithm showed significant fluctuations in the initial stage. However, compared with the traditional SSA method, the PSSSA method has achieved significant improvements in power magnitude and stability. In contrast, the traditional P&O method, due to the lack of appropriate step size adjustment coefficients and flexible migration probabilities, did not approach the maximum power point until 0.18 seconds later and exhibited significant power fluctuations, ultimately outputting a power of approximately 100W.

During the simulation process, the environmental conditions of the photovoltaic array changed from sudden changes in light intensity to the distribution of shadows. The improved PSSSA algorithm was able to converge rapidly and stabilize at around 330W after the sudden change in light intensity, with relatively small waveform oscillations. In contrast, the PSSSA algorithm method under shadow conditions, due to the lack of appropriate light intensity changes and rates, did not approach the maximum power point until 0.1 seconds later. In terms of convergence speed, stability, and search accuracy, it was significantly superior to other algorithms, and the final output power was approximately 290W. The final output power of the improved PSSSA algorithm reached 420W. Therefore, in complex and variable external environments, the refined hierarchical population algorithm demonstrates superior dynamic response capabilities.

## Conclusion

Based on the above research results, a new method combining the intelligent fusion algorithm with maximum power point tracking (MPPT) was proposed. This strategy generates an evenly distributed initial population through a fine hierarchical sequence, uses the population distribution center to guide the population for global exploration, and splits the two main populations for easier management. At the same time, it reorganizes the population positions of each layer to enable each layer to independently optimize, making the entire population more capable of collaborating. Additionally, strategies such as boundary processing functions and power restart mechanisms were introduced to update the population positions within the proposed PSSSA framework. Simulation experiments and laboratory platform analyses demonstrated that the proposed method effectively improved the system performance under various operating conditions, and the following main conclusions were drawn:

1) By generating an evenly distributed initial population through a refined hierarchical sequence, and designing a collaborative optimization method for sparrows and particle groups, the algorithm’s compatibility is enhanced, facilitating the collaboration of the entire population and improving the diversity of the population. In a light mutation environment, the convergence speed of this algorithm is approximately 14% faster compared to the SSA algorithm.
2) When using the PSSSA algorithm for MPPT, compared to the PSO, SSA and P&O algorithms, in the uniform illumination environment, the maximum power point is stable at 310W. The convergence speed and tracking accuracy are both the best. Compared with the PSO algorithm, the power fluctuation is reduced to within 0.1 seconds.
3) The proposed algorithm can quickly and accurately track the new maximum power point even in shadow conditions. It can accurately track the maximum power point within 0.1 seconds. Its tracking stability is significantly better than the other three comparison algorithms. Compared with the SSA algorithm, the size of MPP has increased by approximately 61%, demonstrating higher robustness.

Symbol Explanation Table

The following symbols are used in this manuscript

**Table:** 

Title	Implication
k	Boltzmann constant
T	absolute temperature
q	unit charge quantity
$I_{ph}$	photocurrent
$I_{\text{0}}$	current flowing through the diode
$G_{worst}$	worst position of the sparrow
$S_{best}$	best position of the producer
$G_{best}$	current global optimal position
$p_{id}$	individual in the PSSSA population
$p_{gd}$	global optimal position of the PSSSA population
L	1 × d matrix
$R_{sh}$	parallel resistor
$R_{s}$	series resistor
ω	weight coefficient
H	standard solar irradiance intensity

## Supporting information

S1 File Minimal data set. (PDF)

S2 File The data used in the article. (ZIP)
