# Supplementary material for: Research on the application of refinement hierarchical population algorithm in MPPT of photovoltaic arrays
Source: PLoS One. 2026 Feb 18;21(2):e0342496. doi: 10.1371/journal.pone.0342496 (PMC12915925; doi:10.1371/journal.pone.0342496)
Supplement: S1 File — (PDF) [file pone.0342496.s001.pdf]

```
U=u+1
If (u==6)
   u=1
end
If (u==1)
   D=dc(u)
   dcurrent=D
   counter=1
   return
elseif (u==2)
   D=dc(u)
   dcurrent=D
   counter=1
   return
elseif (u==3)
   D=dc(u)
   dcurrent=D
   counter=1
   return
elseif (u==4)
   D=dc(u)
   dcurrent=D
   counter=1
   return
elseif (u==5)
   [m, i]=max (p)
   gbest=pbest(i)
   D=gbest
   dcurrent=D
   Counter=1
   V (1) =updatevelocity (v (1), pbest(1), dc (1), gbest )
   V (2) =updatevelocity (v (2), pbest(2), dc (2), gbest )
   V (3) =updatevelocity (v (3), pbest(3), dc (3), gbest )
   V (4) =updatevelocity (v (4), pbest(4), dc (4), gbest )
   return
else
```
